# Anatomical Considerations on Surgical Anatomy of the Carotid Bifurcation

**DOI:** 10.1155/2016/6907472

**Published:** 2016-03-07

**Authors:** Adamantios Michalinos, Markos Chatzimarkos, Nikolaos Arkadopoulos, Michail Safioleas, Theodore Troupis

**Affiliations:** ^1^Department of Anatomy, Faculty of Medicine, National and Kapodistrian University of Athens, 17 Mikras Asias Street, Goudi, 15771 Athens, Greece; ^2^3rd Department of Surgery, Attikon University Hospital, Faculty of Medicine, National and Kapodistrian University of Athens, 12243 Athens, Greece

## Abstract

Surgical anatomy of carotid bifurcation is of unique importance for numerous medical specialties. Despite extensive research, many aspects such as precise height of carotid bifurcation, micrometric values of carotid arteries and their branches as their diameter, length, and degree of tortuosity, and variations of proximal external carotid artery branches are undetermined. Furthermore carotid bifurcation is involved in many pathologic processes, atheromatous disease being the commonest. Carotid atheromatous disease is a major predisposing factor for disabling and possibly fatal strokes with geometry of carotid bifurcation playing an important role in its natural history. Consequently detailed knowledge of various anatomic parameters is of paramount importance not only for understanding of the disease but also for design of surgical treatment, especially selection between carotid endarterectomy and carotid stenting. Carotid bifurcation paragangliomas constitute unique tumors with diagnostic accuracy, treatment design, and success of operative intervention dependent on precise knowledge of anatomy. Considering those, it becomes clear that selection and application of proper surgical therapy should consider anatomical details. Further research might ameliorate available treatment options or even lead to innovative ones.

## 1. Introduction

Arterial vascularization of the head and neck area derives from common carotid artery (CCA), vertebral arteries and CCA's branches, external carotid artery (ECA), and internal carotid artery (ICA). The CCA ascends until a level defined by C4 vertebra posteriorly and upper border of thyroid cartilage (TC) anteriorly. There it enlarges into carotid sinus before bifurcating into ECA and ICA. The carotid bifurcation (CB) is an anatomically and surgically important landmark as it is involved in a variety of physiological and pathological processes.

The height of the carotid bifurcation (HCB) is classically defined in relation with vertebral levels and is highly variable across literature. This definition is impractical during an operation as neither the patient is placed in anatomic position nor the vertebrae are accessible. Instead definitions of the HCB in relation with anterior anatomic landmarks are more useful. Extremes of the HCB, that is, the “high” and “low” CB, are of special importance as they can alter the appropriate surgical technique, including selection between carotid endarterectomy and carotid stenting [1, 2]. Finally geometry of CB is a determinant of local blood hemodynamic and wall shear stress, commencing or promoting the process of atherogenesis [3–5].

The ECA right after CB starts giving off its first branches, namely, superior thyroid artery (STA), lingual artery (LA), and facial artery (FA) anteriorly while posterior branches are ascending pharyngeal artery (APA) and occipital artery. Typology and morphometry of those branches are highly variable as anatomic variations are common in the area. Evolvement of intravascular treatments, including embolization and chemoembolization for head and neck tumors, has renewed interest in anatomic variations of the area as they became clinically important.

The CB contains baroceptors able to detect acute changes in arterial pressure alongside chemoceptors able to detect acute changes in arterial oxygen. Those receptors communicate with brainstem and through reflexes regulate homeostasis of these vital parameters [6]. Surgical denervation of CB is a treatment of carotid sinus syndrome [7].

Embryologic origin of the carotid system is not completely clarified. The ICA and the CB develop from 3rd aortic arch while the ECA develops from 2nd aortic arch. From this point of view the ICA should be considered continuation of the CCA while the ECA its branch. Origin of the ECA branches interacts with development of the corresponding vascularized areas. Rare cases of nonbifurcating carotid artery with origin of ICA found at C2 or C1 level and branches of ECA originating from nonbifurcating carotid artery indicate that persistence of primitive hyoid-stapedial system can substitute normal development in case of embryologic arrest. Notably those variations can go unnoticed through lifetime as they are asymptomatic and their connection to diseases (e.g., atherosclerosis and stroke) is unclear [8]. Different and largely unknown mechanisms including duplication or regression of primitive vessels have been proposed to explain anatomical variability in the area [8].

Depiction on HCB and geometry is possible with various modalities such as computerized tomographic angiography, magnetic resonance angiography, and ultrasound [9]. Selection should be based on criteria like its applicability for the speculated disease, availability, and patient-specific characteristics (e.g., allergy at ionizing contrast, metal implants) with 3-dimensional computerized tomography imaging being the gold standard as it provides information on 3-dimensional geometry and guides surgical approach [10, 11].

Although this overview is probably sufficient for most clinical purposes, many anatomic features of carotid arterial system are of particular surgical importance. Anatomic characteristics of interest are the HCB, anatomic variations of the anterior branches of the ECA, morphometric values of the CCA, ICA, and ECA and detailed anatomy of the carotid sinus. Those interact with many pathological mechanisms and their surgical management including carotid atheromatosis and carotid endarterectomy, surgical oncology of head and neck tumors, carotid stenting, and carotid tumors.

## 2. Material and Methods

An electronic bibliographic search was conducted in Medline Embase, Cinahl, and Cochrane Library for studies on CB anatomy. Terms used were “carotid bifurcation”, “external carotid artery branches”, “carotid body”, and “carotid sinus”. Articles not referring to humans or in other language than English were excluded. Results were hand-searched and selected appropriately with reference to their correspondence with the aforementioned keywords. Moreover, literature of selected articles was further hand-searched for relevant publications.

## 3. Discussion

### 3.1. Height of the Carotid Bifurcation

The HCB is usually defined in relation with bony or cartilaginous structures of the neck, that is, cervical vertebrae posteriorly and hyoid bone (HB) and TC anteriorly.

Most textbook position the CB at C3/4 intervertebral disk level or the superior border of the TC. This definition is of limited surgical use because those measurements are usually performed at anatomic position or “Frankfurt plane” [1] while most head and neck operations are performed with the head in extension. Furthermore vertebrae are not accessible during most carotid operations. As Mirjalili et al. stated [1] correspondence between anatomic position of anterior and posterior landmarks is not absolute: HB can be located anywhere between upper C3 and C5/6 intervertebral disk as can TC. Yet a more important but less investigated anatomic landmark is the angle of mandible (AM) because it is a significant barrier to surgical access to the CB. This has been investigated recently by McNamara et al. [12] who measured middle distance between the CB and the AM at 25 mm. Ozgur et al. [13] performed detailed measurement of the CB from different landmarks and found distance between the CB and the TC at 9.8 ± 6.7 mm, CB and HB at 8.7 ± 6 mm, and CB and gonion at 36.2 ± 9.4 mm. The HCB is not affected by age or gender, yet there is marked asymmetry between sides ranging between 50 and 75% across various studies [1, 6, 14, 15].

Table 1 encompasses larger studies investigating HCB in relation to the aforementioned landmarks.

#### 3.1.1. The “High” and “Low” Bifurcation

The terms high and low bifurcation are commonly used in medical literature and routine clinical practice but they lack a precise anatomic definition. The HCB can be determined only radiographically; no reliable clinical signs exist [14]. Many authors define high bifurcation as higher than C3/4 intervertebral disk while others as higher than greater horn of HB. McNamara et al. [12] instead define high bifurcation as the one lying in 1st quartile of HCB distribution. As this is a statistical definition, authors correlated this distance with a distance from a known and steady anatomical point. Distance from mastoid process is a reliable indicator of high carotid bifurcation as is the CB position above the HB. Instead classical definition of the HCB in concordance with cervical vertebrae presents mild only correlation and utility.

Definition of high bifurcation is surgical and implies difficult surgical access due to bony elements, specifically the AM. Current anatomic definitions of high CB are of limited use [16]. Anatomic studies in the proper position, that is, with the head in extension and contralateral turn, might provide more evidence.

Origin of the ECA from top of 3rd aortic arch or directly from dorsal aorta and origin of ICA from 2nd aortic arch concomitant with ECA formation from small canals are proposed embryologic explanations for high bifurcation [6, 17, 18]. High bifurcation is commoner in Japanese [19], females [15], and at the left side [6]. Woldeyes [20] reported an unusually high incidence of high bifurcation in an Ethiopian population, indicating a genetic component.

High bifurcation is of utmost surgical importance for operations in head and neck area. Commonest operation in area is carotid endarterectomy. High bifurcation is a predisposing factor for surgical complications, including injury of cranial nerves. Hypoglossal and marginal mandibular nerves are nerves most commonly injured at a rate of 5.2% [12, 21]. Notably height of hypoglossal nerve is not correlated with HCB; thus in case of a high CB distance between them is smaller [15].

Accordingly sophisticated yet copious and potentially dangerous surgical maneuvers have been introduced to solve the problem of high bifurcation. Main techniques are styloidectomy [22], mandibulotomy [23], and mandibular subluxation [24]. Unfortunately those techniques present important complications including bone infection, facial palsy, nonunion of the mandible, and difficulty with mastication [24]. Other maneuvers such as nasotracheal instead of orotracheal intubation did not produce satisfactory results [25].

A possible solution to the problem of high CB is carotid artery stenting instead of carotid endarterectomy. A number of large studies have failed to prove noninferiority of carotid artery stenting to carotid endarterectomy both for symptomatic carotid atheromatosis and asymptomatic carotid artery stenosis. Carotid stenting presents a higher risk of complications including stroke and myocardial infarction. Current strongest indications for carotid artery stenting instead of carotid endarterectomy are a high carotid bifurcation, restenosis after previous endarterectomy, operated neck for other reason, and contralateral occlusion of the CCA or the ICA [9, 26–28], as stated in most recent AHA/ASA guidelines for stroke prevention (Class IIa, Level of evidence B) [2].

Instead of its counterpart, low CB is considered surgically favorable and has not received special attention with only sparse reports on its implications on surgical operations, including anterior cervical discectomy [29]. Low CB is rare with an incidence of 3.75% [30] and 7.5% [31] across two studies. A proposed embryologic explanation for low bifurcation is ECA origin from low in aortic arch [6]. Very rare reports of double communication between ECA and ICA (“island formation”) propose persistence of both 2nd and 3rd aortic arch as possible explanations [17]. Extreme cases of intrathoracic level of CB have been reported [32].

### 3.2. Diameter and Tortuosity of Carotid Arteries System

Natural history of carotid atheromatous disease is affected by local flow parameters which are in turn affected by carotid artery system geometry [33]. Any bifurcation acts naturally as a point of disturbance of local flow fields [34]. Tortuous and angled vessels are more prone to plaque development due to turbulous flow [35]. Ozgur et al. [13] performed detailed anatomical studies and calculated the CCA, ICA, ECA, and CB diameter at 8.1 ± 2.24, 6.1 ± 1.3, 6.6 ± 1.3, and 12.79 ± 0.87 mm, respectively. However those measurements while useful for stent and intravascular catheters design might have limited only importance for carotid atheromatous disease investigations. Goubergrits et al. [34] measured the caliper of the CCA at 6.61 mm, ICA at 7.38 mm, and ECA at 5.98 mm. Notably authors consider those vessels as elliptical rather than round shaped and thus those measures correspond to major axes. They also found that the CB angle, that is, the angle between ICA and ECA, is 51 degrees in females and 67 degrees in males and that the CB angle is correlated to atherosclerosis existence. Various authors have calculated ICA/ECA, ICA/CCA, and ECA/CCA diameter and flow ratios which are predictive of disturbed flow in CB. An overview of these researches is presented in Table 2. Still value of these studies is doubtful since, as stated by the authors, no differences are found between atheromatous and nonatheromatous CCAs [3, 4]. Given inconclusiveness of current methods, Miralles et al. [36] proposed a method of 3-dimensional volumetric assessment of CB. Differences in endovascular volumetry are correlated with progression of atheromatous plaque, while Thomas et al. [37] stated that increase in ICA/CCA, ECA/CCA, and ECA/ICA angle and increase in vessels tortuosity are early markers of carotid atheromatous disease.

Tortuosity of CCA has also been subjected to research. According to Lo et al. [14] only 63% of the CCAs follow a straight course while 26% are curved and 6% are kinked or coiled. A rare case of bilateral kinking of the ICA and coiling with extreme tortuosity of the ECA has been reported by Cvetko [38]. Authors also comment on histologic changes seen in this rare case, that is, metaplasia of tunica media, reduction of elastic fibers, and muscular cells and consequent wall thinness. Those changes are often seen in atheromatous carotid arteries. Marked tortuosity of the CCA and marked tortuosity of the ICA are also strong indications for carotid artery stenting instead of carotid endarterectomy [27].

Relative positions of the ICA and the ECA have also been investigated. While classical textbook describes ECA as being anteromedial to ICA, their position can be reversed between 1.7 and 7.5% of cases [13, 15, 39].

### 3.3. Proximal Branches to the Carotid Bifurcation

According to classical textbooks first branch of the ECA is the STA. After its origin STA follows a descending oblique course before entering at superior lobe of thyroid gland. Branches of the STA are infrahyoid, superior laryngeal, and sternocleidomastoid branches. Second branch of the ECA is the APA. The APA after its origin from posterior wall of ECA ascends giving vascularization to pharynx. Third branch of the ECA is the LA. The LA originates from the ECA at the level of the posterior tubercle of the HB. In its hypocarotid segment, the artery runs obliquely upward, forward, and medially and gives off muscular branches that attach it to the middle constrictor of the pharynx and a fine suprahyoid branch. Those branches are in close companion with CB and thus of importance during various surgical procedures.

Origin of the STA from the ECA has been disputed by a number of publications. Natsis et al. [40] state that since the CCA and CB originate from 3rd aortic arch while the ECA from 1st aortic branch, the CCA and the CB should be considered an anatomical and physiological unity, the ICA typical continuation of CCA and the ECA a branch of the CCA. Branches originating from the CB should be considered branches of the CCA rather than branches of the ECA. In the same paper they stated that the STA originates from the CCA-CB at 61% while it originates from the ECA only at 39%. Considering this, many authors [6, 10, 13, 14, 39, 41–44] have found origin of the STA from the CCA-CB in 50–75%. According to a meta-analysis from Toni et al. [45] origin of the STA from the ECA is commoner at right side and in Caucasians. Distance of the STA from the CB has been measured in various studies. Results are presented in Table 3.

According to the radiographic study by Small et al. [46] the APA originates from the CCA or the CB at 6.5% and according to Cappabianca et al. [10] at 9.6%. LA origin from the CCA is rare and <1% and is mainly found in form of case reports or combined with other anatomic variations [47].

Asymmetry between branching patterns is high, between 50 and 75% [10, 39, 40, 48]. Origin of common trunks from CB including Lingual-Facial Trunk [10, 49], Thyrolingual Trunk [50], and Thyrolingual-Facial Trunk [40] has been reported. Other uncommon trunks such as occipitoauricular have also been reported [19]. Notably in cases of high bifurcation origin of anterior branches from the CCA or the CB and formation of common trunk is commoner while their origins' distance is smaller [17, 42]. An extreme case reported by Gluncic et al. [18] consisted of a very high bifurcation with origin of the STA, LA, and APA and occipital artery directly from the CCA while the ECA was hypoplastic. The opposite also stands true, common trunks formation is rare, and branches origin is larger in cases of low bifurcation [15].

#### 3.3.1. Clinical Importance of Anterior Branches

Detailed morphometry of the CB and front branches of the ECA is implicated in surgical management, chemoembolization, and large defects restoration during management of head and neck tumors. Selective and superselective chemoembolization of head and neck tumors, especially laryngeal and hypopharyngeal, is of proven oncologic value but catheterization of the ECA branches demands caution, expertise, and knowledge of their anatomic variability [42]. Usual feeding vessels are the STA and LA but almost half of these tumors feed from more than one vessel sometimes contralaterally from their site and thus multiple catheterizations might be necessary [44]. Existence of common trunks if unrecognized can cause diffusion of chemotherapeutic agents in undesired sites while small vessels might be inadequate for catheterization leading to catheterization accidents (rupture, thrombosis) or infusion of chemotherapeutic agents to undesired sites through disturbed flow.

Head and neck tumors excision can lead to large defects with functional and aesthetical disability. Various flaps such as the pedicled nasogenial flap, the islet flaps with subcutaneous pedicles, the rotation flaps of Mustardé type, and Estlander's flap have been invented for coverage [51, 52]. Microvascular anastomosis with front branches of the ECA, especially the STA and LA, is feasible but improper selection might lead to an insufficient microvascular anastomosis. Failure of the anastomosis can cause flap necrosis, shrinkage, or hemorrhage. Proper selection based on vessels' sufficiency in terms of diameter and length is of critical importance.

Finally during carotid endarterectomy ligation of front branches of ECA for mobilization of CB is feasible due to rich anastomotic vasculature in the area. Carotid endarterectomy can be performed with maintenance of front branches [53] and this is important in rare cases of unsafe ligation of the STA or LA that could cause ischemia of larynx or tongue (e.g., operated contralateral side).

### 3.4. Carotid Sinus Syndrome

The CB maintains distinct role in arterial pressure regulation through baroceptors located in its adventitia. Those are capable of detecting sudden rise of arterial pressure and through baroceptors reflex provide negative feedback and reregulate arterial pressure. Afferent branch of this reflex is glossopharyngeal nerve (IX) and efferent is vagus nerve (X). Arterial pressure control occurs through downregulation of sympathetic activity [54].

Toorop et al. [7] performed analytical dissection on the afferent limb of the baroceptors reflex. They investigated that nerve fibers originating directly from carotid bifurcation form the carotid sinus nerve (CSN) (Hering or Castro nerve). Then the CSN ascends in close proximity with the ICA and joins the hypopharyngeal nerve. They also proved that anatomy and position of the nerve are unstable often presenting 2 branches and communications with other cranial nerves, especially vagus nerve.

Carotid sinus syndrome manifests with improper activation of baroceptors reflexes leading to sudden fall in arterial pressure and cardiac rhythm (asystole) and probably to loss of consciousness. While physiology of carotid sinus syndrome is not yet completely clarified, it is believed that improper activation begins at baroceptors at carotid sinus. Improper activation is usually unilateral (55% on right side and 21% on the left) [55]. Current treatment of carotid sinus syndrome consists of medical therapy and electric stimulation of the heart through a pacemaker [55, 56] while surgical denervation of carotid sinus has limited efficacy, probably due to rich anastomotic plexus in the area. Detailed anatomic studies in the area could reveal more details on the subject and thus ameliorate surgical therapy leading to higher success rates [7].

### 3.5. Carotid Body Tumors

Carotid body tumors are paragangliomas originating from neural crest-derived paraganglionic neuroectodermal cells in the CB [57]. They are rare tumors with incidence <1/30,000 and a female predilection (M/F = 2) [58]. They usually occur during 3rd and 4th decade of life. They are bilateral in approximately 20% of the cases [59]. Clinically they manifest as enlargement in the neck area. They produce symptoms through mass-effect pressure or by directly invading structures in neck area, such as hoarseness due to infiltration of superior laryngeal nerve [59]. Their malignant potential is uncertain with histologic examination only and is usually confirmed by presence of metastatic disease [60]. They usually metastasize in lymph nodes and bones and much rarer in liver or viscera [57].

Etiology of those tumors is unclear. Due to their rarity, no predisposing factors have been recognized. Most of them are sporadic and about 10% of them are familial. Interestingly they are more frequent in population living in high altitudes and this has suggested that they represent an extreme form of hyperplasia of carotid sinus cells due to chronic hypoxia [59, 61].

Treatment of these tumors is surgical excision. Removal of the primary tumor demands meticulous dissection of the CB and the surrounding anatomic structures. Shamblin classification in type I (not surrounding vessel), II (partially surrounding vessel), or III (encircling vessel) is of limited use in predicting need of vascular reconstruction of CB [58] while more important is vessel infiltration by the tumor. Surgical therapy is also beneficial for metastatic disease while bone metastasis or inoperable disease is treated with radiotherapy and medical therapy [57]. Morbidity associated with surgical excision is high and is connected with cranial nerves injury due to extensive dissection, stroke due to temporal occlusion of the vessel, and hemorrhage due to injury of the carotid arteries during dissection. Different maneuvers have been tested including intraoperative shunts, preoperative embolization of the carotids, and radiotherapy yet due to rarity of those tumors results remain controversial. Preoperative definition of the anatomy with all possible radiological means, including computerized tomographic arteriography, seems a cardinal factor for success. Disease progression is usually slow allowing a good prognosis [61].

## 4. Conclusions

Surgical anatomy of the CB area is complex but important for many different clinical and surgical applications. Anatomically it should not be seen as the bifurcation point between ICA and ECA only but rather in conjunction with other important anatomic structures in the area, including cranial nerves. Many questions on its detailed anatomy are not completely clarified including precise HCB, morphometry of the ICA and ECA, and tortuosity of the CCA. Its embryological origin, although based on solid embryologic theories, is not completely understood.

The CB is the operative target in the relative common carotid atheromatosis disease and other rarer diseases like carotid body tumors and carotid aneurysms. Pathogenesis of atheromatosis can be further explained by detailed morphometry of CB, as hemodynamic is affected by morphometric values. Also clarification of anatomy can alter surgical interventions and help in distinguishing subgroups of patients necessitating special interventions. Detailed anatomic knowledge of ECA branches is important for radiologic interventions including embolization and chemoembolization for head and neck tumors. Treatment of carotid sinus syndrome and carotid body tumors also implies thorough understanding and knowledge of CB anatomy.

## Figures and Tables

**Table 1:** HCB in relation to different anatomic landmarks.

Author	Study type/sample	HCB (anterior)	HCB (posterior)
Espalieu et al. [42], 1986	Cadaveric (36) Angiographic (50)		C2/3: 6%, C3/4: 25%, C4: 65% C4-C5: 4%

Lučev et al. [43], 2000	Cadaveric (20)	Superior level of HB: 12.5% Inferior level of HB: 10% Superior level of TC: 50% Inferior level of TC: 5%	

Zümre et al. [41], 2005	Cadaveric (40)		C3 (l): 60% (r): 55% C4 (l): 45% (r): 35% C5 (l): 0% (r): 10%

Ito et al. [15], 2006	Cadaveric (40)		C2, C2/3, C3, C3/4: 31% C4: 58% C4/5: 11%

Lo et al. [14], 2006	Cadaveric (36)	Greater horn of HB: 15% Body of HB: 40% Superior border of TC: 39% Body of TC: 6%	

Pai et al. [62], 2007	Cadaveric (95)		C2 (l): 10% (r): 9% C3 (l): 50% (r): 55% C4 (l), 40% (r): 35% C5 (l): 0% (r): 1%

Klosek and Rungruang [6], 2008	Cadaveric (43)		C2/3: 2.3% C3: 10.4% C3/4: 20.9% C4: 30.2% C4/5: 16.3% C5: 8.1%

Al-Rafiah et al. [39], 2011	Cadaveric (30)	Higher than HB: 3.3% HB: 25% Between HB and TC: 18.3% Superior level of TC: 48.3% Lower than TC: 5%	

McNamara et al. [12], 2015	Angiographic (76)	Above AM: 0.7% Same level with AM Below AM: 95.7% Above HB: 62.9% Same level with HB: 26.4% Below HB: 10.7% Above TC: 79.3% Same level with TC: 16.4% Below TC: 4.3%	

HB: hyoid bone; TC: thyroid cartilage; AM: angle of mandible; HCB: height of carotid bifurcation.

**Table 2:** Diameter ratios of the carotid arterial system.

Author	Study type	ICA/CCA	ECA/CCA	ICA/ECA	Inflow/outflow area
Goubergrits et al. [34], 2002	Cadaveric (86)	1.1 (0.63–1.47)			1.78 (0.67–3.21)
Schulz and Rothwell [3], 2001	Angiographic (5395)	0.63 (0.44–0.86)	0.55 (0.34–0.80)	0.88 (0.55–1.33)	0.73 (0.38–1.28)
Şehirli et al. [4], 2005	Cadaveric (20)	0.71 ± 0.13	0.78 ± 0.12	0.93 ± 0.16	1.14 ± 0.28.
Thomas et al. [35], 2005	MRA (50)	0.81 ± 0.06	0.81 ± 0.06		
Ozgur et al. [13], 2008	Cadaveric (20)	0.98	0.85	0.86	

MRA: magnetic resonance angiography; ICA: internal carotid artery; CCA: common carotid artery; ECA: external carotid artery.

**Table 3:** Distance of STA from CB.

Author	Study type	Distance STA-CB	Origin of STA from CCA-CB
Espalieu et al. [42], 1986	Cadaveric (36) Angiographic (50)	1.5–8 mm	55%
Lučev et al. [43], 2000	Cadaveric (20)	2–10 mm	70%
Lo et al. [14], 2006	Cadaveric (65)	5.9 mm^*∗*^ 3 mm^†^	53.8%
Klosek and Rungruang [6], 2008	Cadaveric (43)	6.5 ± 3.2 mm^*∗*^ 7.1 ± 0.3 mm^†^	33.3%
Ozgur et al. [13], 2008	Cadaveric (20)	3.3 ± 4.3 mm	75%
Vázquez et al. [63], 2009	Cadaveric (207)	1–21 mm^*∗*^ 1–15 mm^†^	76%
Al-Rafiah et al. [39], 2011	Cadaveric (30)	4–11 mm	94%

^*∗*^STA origin from ECA, ^†^STA origin from CCA.

STA: superior thyroid artery, CCA: common carotid artery, CB: carotid bifurcation.

## References

[1] Mirjalili S. A., McFadden S. L., Buckenham T., Stringer M. D. (2012). Vertebral levels of key landmarks in the neck. *Clinical Anatomy*.

[2] Kernan W. N., Ovbiagele B., Black H. R. (2014). Guidelines for the prevention of stroke in patients with stroke and transient ischemic attack: a guideline for healthcare professionals from the American Heart Association/American Stroke Association. *Stroke*.

[3] Schulz U. G. R., Rothwell P. M. (2001). Major variation in carotid bifurcation anatomy: a possible risk factor for plaque development?. *Stroke*.

[4] Şehirli Ü. S., Yalin A., Tulay C. M., Cakmak Y. O., Gürdal E. (2005). The diameters of common carotid artery and its branches in newborns. *Surgical and Radiologic Anatomy*.

[5] Ardakani S., Jafarnejad M., Firrozabadi B., Saidi M. (2010). Investigation of wall shear stress related factors in realistic carotid bifurcation geometries and different flow condition. *Scientia Iranica Transaction B, Mechanical Engineering*.

[6] Klosek S. K., Rungruang T. (2008). Topography of carotid bifurcation: considerations for neck examination. *Surgical and Radiologic Anatomy*.

[7] Toorop R. J., Scheltinga M. R., Moll F. L., Bleys R. L. (2009). Anatomy of the carotid sinus nerve and surgical implications in carotid sinus syndrome. *Journal of Vascular Surgery*.

[8] Kim C. H., Cho Y. D., Kang H. (2015). Anomalous external carotid artery-internal carotid artery anastomosis in two patients with proximal internal carotid arterial remnants. *Korean Journal of Radiology*.

[9] Ashrafian H. (2007). Anatomically specific clinical examination of the carotid arterial tree. *Anatomical Science International*.

[10] Cappabianca S., Scuotto A., Iaselli F. (2012). Computed tomography and magnetic resonance angiography in the evaluation of aberrant origin of the external carotid artery branches. *Surgical and Radiologic Anatomy*.

[11] Vaiman M., Bekerman I. (2015). Preoperative detection of anomalies of carotid arteries in the neck surgery. *European Archives of Oto-Rhino-Laryngology*.

[12] McNamara J. R., Fulton G. J., Manning B. J. (2015). Three-dimensional computed tomographic reconstruction of the carotid artery: identifying high bifurcation. *European Journal of Vascular and Endovascular Surgery*.

[13] Ozgur Z., Govsa F., Ozgur T. (2008). Anatomic evaluation of the carotid artery bifurcation in cadavers: implications for open and endovascular therapy. *Surgical and Radiologic Anatomy*.

[14] Lo A., Oehley M., Bartlett A., Adams D., Blyth P., Al-Ali S. (2006). Anatomical variations of the common carotid artery bifurcation. *ANZ Journal of Surgery*.

[15] Ito H., Mataga I., Kageyama I., Kobayashi K. (2006). Clinical anatomy in the neck region—the position of external and internal carotid arteries may be reversed. *Okajimas Folia Anatomica Japonica*.

[16] Chakfé N., Ohana M., Georg Y. (2015). Commentary on “Three-dimensional CT reconstruction of the carotid artery: identifying the high bifurcation”. *European Journal of Vascular and Endovascular Surgery*.

[17] Iimura A., Oguchi T., Yamazaki Y. (2010). Anomalous bifurcation and island formation of the carotid artery. *Okajimas Folia Anatomica Japonica*.

[18] Gluncic V., Petanjek Z., Marusic A., Gluncic I. (2001). High bifurcation of common carotid artery, anomalous origin of ascending pharyngeal artery and anomalous branching pattern of external carotid artery. *Surgical and Radiologic Anatomy*.

[19] Hayashi N., Hori E., Ohtani Y., Ohtani O., Kuwayama N., Endo S. (2005). Surgical anatomy of the cervical carotid artery for carotid endarterectomy. *Neurologia Medico-Chirurgica*.

[20] Woldeyes D. H. (2014). Anatomical variations of the common carotid artery bifurcations in relation to the cervical vertebrae in Ethiopia. *Anatomy & Physiology*.

[21] Assadian A., Senekowitsch C., Pfaffelmeyer N., Assadian O., Ptakovsky H., Hagmüller G. W. (2004). Incidence of cranial nerve injuries after carotid eversion endarterectomy with a transverse skin incision under regional anaesthesia. *European Journal of Vascular and Endovascular Surgery*.

[22] Mock C. N., Lilly M. P., McRae R. G., Carney W. I. (1991). Selection of the approach to the distal internal carotid artery from the second cervical vertebra to the base of the skull. *Journal of Vascular Surgery*.

[23] Fortes F. S. G., da Silva E. S., Sennes L. U. (2007). Mandibular subluxation for distal cervical exposure of the internal carotid artery. *Laryngoscope*.

[24] Yoshino M., Fukumoto H., Mizutani T., Yuyama R., Hara T. (2010). Mandibular subluxation stabilized by mouthpiece for distal internal carotid artery exposure in carotid endarterectomy. *Journal of Vascular Surgery*.

[25] Foreman P. M., Harrigan M. R., Griessenauer C. J., Loukas M., Tubbs R. S. (2016). Access to the carotid artery bifurcation: cadaveric study with application to nasotracheal intubation as a technique to improve access to a high carotid artery bifurcation. *British Journal of Neurosurgery*.

[26] Kolkert J. L., Meerwaldt R., Geelkerken R. H., Zeebregts C. J. (2015). Endarterectomy or carotid artery stenting: the quest continues part two. *American Journal of Surgery*.

[27] Dangas G., Laird J. R., Mehran R. (2001). Carotid artery stenting in patients with high-risk anatomy for carotid endarterectomy. *Journal of Endovascular Therapy*.

[28] Rockman C., Loh S. (2011). Carotid endarterectomy: still the standard of care for carotid bifurcation disease. *Seminars in Vascular Surgery*.

[29] Gulsen S., Caner H., Altinors N. (2009). An anatomical variant: low-lying bifurcation of the common carotid artery, and its surgical implications in anterior cervical discectomy. *Journal of Korean Neurosurgical Society*.

[30] Anangwe D., Saidi H., Ogeng'o J., Awori K. O. (2008). Anatomical variations of the carotid arteries in adult Kenyans. *East African Medical Journal*.

[31] Vatsala A., Ajay K. (2014). A study of anatomical variations of the common carotid arteries: a cadaveric study. *International Journal of Anatomy and Research*.

[32] Gomez C. K., Arnuk O. J. (2013). Intrathoracic bifurcation of the right common carotid artery. *BMJ Case Reports*.

[33] Lee S.-W., Antiga L., Spence J. D., Steinman D. A. (2008). Geometry of the carotid bifurcation predicts its exposure to disturbed flow. *Stroke*.

[34] Goubergrits L., Affeld K., Fernandez-Britto J., Falcon L. (2002). Geometry of the human common carotid artery. A vessel cast study of 86 specimens. *Pathology Research and Practice*.

[35] Thomas J. B., Antiga L., Che S. L. (2005). Variation in the carotid bifurcation geometry of young versus older adults: implications for geometric risk of atherosclerosis. *Stroke*.

[36] Miralles M., Arrébola M., Bruguer S., Lago A., Lara R. (2015). Volumetric assessment of the carotid bifurcation: an alternative concept to stenosis grading. *Annals of Vascular Surgery*.

[37] Thomas B., Che S., Milner J. Geometric characterization of the normal and mildly diseased human carotid bifurcation.

[38] Cvetko E. (2014). Concurrence of bilateral kinking of the extracranial part of the internal carotid artery with coiling and tortuosity of the external carotid artery—a case report. *Romanian Journal of Morphology and Embryology*.

[39] Al-Rafiah A., El-Haggagy A. A., Aal I. H. A., Zaki A. I. (2011). Anatomical study of the carotid bifurcation and origin variations of the ascending pharyngeal and superior thyroid arteries. *Folia Morphologica*.

[40] Natsis K., Raikos A., Foundos I., Noussios G., Lazaridis N., Njau S. N. (2011). Superior thyroid artery origin in Caucasian Greeks: a new classification proposal and review of the literature. *Clinical Anatomy*.

[41] Zümre Ö., Salbacak A., Çiçekcibaşi A. E., Tuncer I., Seker M. (2005). Investigation of the bifurcation level of the common carotid artery and variations of the branches of the external carotid artery in human fetuses. *Annals of Anatomy*.

[42] Espalieu P., Cottier M., Relave M., Youvarlakis P., Cuilleret J. (1986). Radio-anatomic study of the carotid axis with regard to the implantation of microsurgical vascular anastomoses. *Surgical and Radiologic Anatomy*.

[43] Lučev N., Bobinac D., Marič I., Drešćik I. (2000). Variations of the great arteries in the carotid triangle. *Otolaryngology—Head and Neck Surgery*.

[44] Terayama N., Sanada J., Matsui O. (2006). Feeding artery of laryngeal and hypopharyngeal cancers: Role of the superior thyroid artery in superselective intraarterial chemotherapy. *CardioVascular and Interventional Radiology*.

[45] Toni R., Della Casa C., Castorina S. (2004). A meta-analysis of superior thyroid artery variations in different human groups and their clinical implications. *Annals of Anatomy*.

[46] Small J. E., Harrington J., Watkins E. (2014). Prevalence of arterial branches arising from the extracranial internal carotid artery on CT angiography. *Surgical and Radiologic Anatomy*.

[47] Troupis T., Michalinos A., Dimovelis I., Demesticha T., Vlasis K., Skandalakis P. (2014). Bilateral abnormal origin of the anterior branches of the external carotid artery. *Annals of Vascular Surgery*.

[48] Yonenaga K., Tohnai I., Mitsudo K. (2011). Anatomical study of the external carotid artery and its branches for administration of superselective intra-arterial chemotherapy via the superficial temporal artery. *International Journal of Clinical Oncology*.

[49] Troupis T. G., Dimitroulis D., Paraschos A. (2011). Lingual and facial arteries arising from the external carotid artery in a common trunk. *American Surgeon*.

[50] Lemaire V., Jacquemin G., Medot M., Fissette J. (2001). Thyrolingual trunk arising from the common carotid artery: a case report. *Surgical and Radiologic Anatomy*.

[51] Midy D., Mauruc B., Vergnes P., Caliot P. (1986). A contribution to the study of the facial artery, its branches and anastomoses; application to the anatomic vascular bases of facial flaps. *Surgical and Radiologic Anatomy*.

[52] Loukas M., Hullett J., Louis R. G. (2006). A detailed observation of variations of the facial artery, with emphasis on the superior labial artery. *Surgical and Radiologic Anatomy*.

[53] Chan Y. C., Wong W. H., Cheng S. W. (2013). Successful carotid endarterectomy in a patient with an aberrant branch from the common carotid artery. *Annals of the Royal College of Surgeons of England*.

[54] Oberg P. A., Sjöstrand U. (1971). Studies of blood-pressure regulation. 3. Dynamics of arterial blood pressure on carotid-sinus nerve stimulation. *Acta Physiologica Scandinavica*.

[55] Puggioni E., Guiducci V., Brignole M. (2002). Results and complications of the carotid sinus massage performed according to the ‘method of symptoms’. *American Journal of Cardiology*.

[56] Filippone J. D., Bisognano J. D. (2007). Baroreflex stimulation in the treatment of hypertension. *Current Opinion in Nephrology and Hypertension*.

[57] Mediouni A., Ammari S., Wassef M. (2014). Malignant head/neck paragangliomas. Comparative study. *European Annals of Otorhinolaryngology, Head and Neck Diseases*.

[58] Gad A., Sayed A., Elwan H. (2014). Carotid body tumors: a review of 25 years experience in diagnosis and management of 56 tumors. *Annals of Vascular Diseases*.

[59] Metheetrairut C., Chotikavanich C., Keskool P., Suphaphongs N. (2015). Carotid body tumor: a 25-year experience. *European Archives of Oto-Rhino-Laryngology*.

[60] Lack E. (2007). *Tumors of the Adrenal Glands and Extraadrenal Paraganglia*.

[61] Taha A. Y. (2015). Carotid body tumours: a review. *International Journal of Clinical Medicine*.

[62] Pai M. M., Rajalakshmi R., Latha V. P., Rajanigandha V., D'Costa S., Anu V. R. (2007). Clinically-relevant variations of the carotid arterial system. *Singapore Medical Journal*.

[63] Vázquez T., Cobiella R., Maranillo E. (2009). Anatomical variations of the superior thyroid and superior laryngeal arteries. *Head and Neck*.

